# Seismic refraction investigation of the shallow bedrock in New Qena City, Eastern Desert, Egypt

**DOI:** 10.1038/s41598-025-85949-5

**Published:** 2025-01-21

**Authors:** Ahmed M. Abdelgowad, Assem E. El-Haddad, Mohamed I. Aglan, Ahmed Hamed

**Affiliations:** 1 Geology Department, Faculty of Science, South Valley University, Qena, Egypt; 2 Geology Department, Faculty of Science, Assiut University, Assiut, Egypt; 3 General Petroleum Company, Eastern Desert Fields, Ras Gharib, Egypt; 4 Aswan Regional Earthquake Research Center, National Research Institute of Astronomy and Geophysics (NRIAG), Helwan, Egypt

**Keywords:** Geology, Geophysics

## Abstract

The seismic refraction technique has demonstrated its efficiency as a cost-effective geophysical approach for bedrock investigation, which is very important for major construction projects. In the southern part of New Qena City, in the Eastern Desert of Egypt, construction of many domestic facilities is planned. Therefore, a prior investigation focusing on bedrock is required to validate the site for construction and other projects. In this study, a site investigation of the southern part of New Qena City is conducted using the seismic refraction method to estimate the depth and thickness of the bedrock and to present the subsurface structural features affecting the area. Ten seismic refraction profiles were measured, and the data were used for tomographic inversion. The results revealed a four-layered subsurface, with the bedrock represented by the mudstone unit of the Pliocene Durri Formation. This layer is located at a shallow depth and exhibits relatively low velocities as well as lateral velocity variations. This is attributed to the clay content of the layer, the high degree of fracturing, and the lateral facies variation. Therefore, the layer could undergo geotechnical problems that could affect future construction projects in the area. Ten subsurface faults were also detected in the velocity sections.

## Introduction

The shallow seismic refraction is not only a non-invasive and cost-effective method but has also been used as a powerful geophysical tool in engineering and environmental investigations. The method is now widely used as the main technique for most engineering purposes, including measuring depth to the water table^1–13^, delineating bedrock surface^14–21^, estimating soil type and geotechnical properties^22–32^, mapping sink holes and voids^33–41^, and estimating rock rippability^16,42–46^.

New Qena City was constructed in 2005 east of the Nile River and east of Qena City, and north of the Qena-Safaga Road that is considered the most critical transportation route in the middle Eastern Desert of Egypt. The city occupies the Quaternary sediments that cover the floor of the mouth of Wadi Qena. The area is characterized by low topography and vast plains that are suitable for both civil construction and agriculture. Much of the city has already been built and settled. Additions to the city south of Qena-Safaga Road are still in the planning stages. Therefore, prior information on the subsurface layering and geological conditions is required for further construction south of Qena-Safaga Road.

The area has been investigated geologically and geophysically by numerous scholars^47–61^. These studies focus mainly on the geologic and hydrogeologic setting of the entire area south of Wadi Qena. El-Akraby (1991)^49^, Othman (1993)^50^ and Omran et al. (1995)^51^ pointed out that the shallow structures in this area are mostly fault blocks. Abu El-Ella (2004)^55^ and Elsadek et al. (2019)^61^ stated that Wadi Qena is classified as very dangerous in terms of flash flood impacts as it contributes the largest amounts of sediments during flash floods. Philobbos et al. (2015)^58^ pointed out that the area was a part of a lake depositional system called Qena Lake that were developed in the Neogene through three phases: a lacustrine–alluvial complex phase; creation of another lake (Issawya Lake); and connection of Wadi Qena with the Red Sea basement mountain range. Basher (2003)^54^ and Moubark and Abdelkareem, (2018)^60^ show that the area lies within the zones of high to very high groundwater potential and contains both shallow aquifer at a depth of 10 to 54 m and deeper aquifer at a depth of 50 to 76 m.

In this study, the main concern is placed on the subsurface bedrock layer, which is represented by the Pliocene Durri Formation^62^. In Qena area, the Durri Formation is composed of lacustrine deposits that consist of mudstones, limestones and marls^63,64^. This layer was considered to be problematic due to its clay content, in which might lead to engineering problems such as swelling and shrinkage, which can lead to significant volume changes in the soil, causing differential settlement and structural damage to foundations, pavements, and other infrastructure^65^. Studies by^66–69^ reported damage to infrastructure along Qena-Safaga road due to swelling related to the clay minerals in the Durri Formation. In addition, the layer was also proved to be responsible for the fractures that cut through Qena-Safaga Road from KM 18 to KM 32^57,69,70^. The low shear strength of the clayey layers can lead to slope failures, and increased earth pressure on retaining structures^71^. Clayey soils can undergo significant consolidation over time when subjected to loads, leading to long-term settlement of structures. This is particularly problematic for heavy structures and can result in uneven settlement and cracking^72^.

**Fig. 1:**
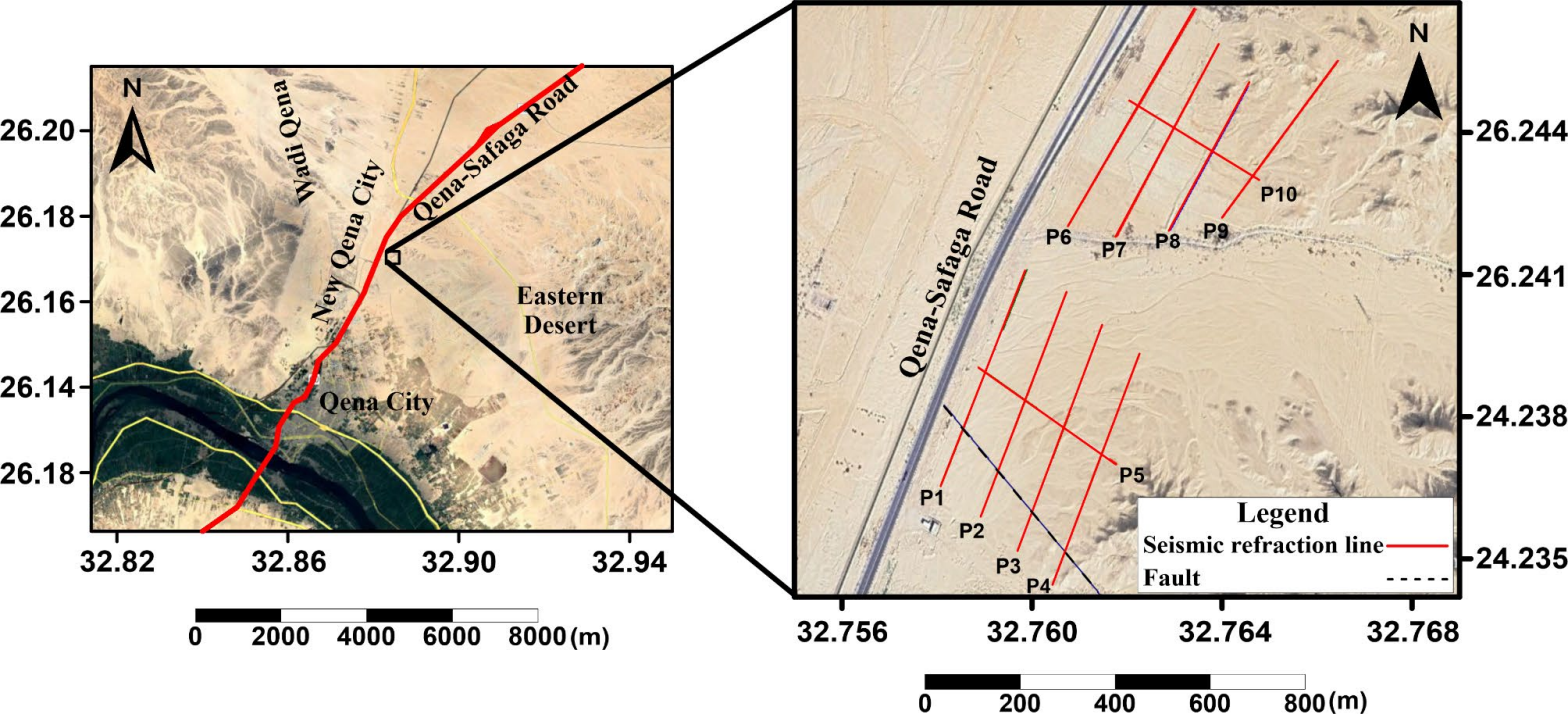
The location map of the study region, which occupies the southern vicinity of New Qena City. This area is positioned to the south of Qena-Safaga Road and situated to the east of the Nile River. The fault trending NW–SE and cut across the western portions of the lines P1 to P4, is detected by the magnetic investigation of^49,52,56,73,74^. [The map is generated by Surfer (Golden Software, LLC), version: 13.6.618^89^].

Due to its importance for the upcoming construction projects in the southern portions of New Qena City, determining depth and thickness of the Durri Formation layer is an essential engineering requirement. In addition, prior information regarding the structural features in the shallow subsurface is also vital. Previous magnetic investigation shows that the region might be impacted by multiple faults^49,52,56,73,74^. These studies confirmed a NW–SE fault running through area, perpendicular to Qena-Safaga Road (Fig. 1). An important objective of this research is to explore this fault and to locate other faults in the shallow layers in this area.

This study provides an investigation of the southern portion of New Qena City using seismic refraction technique. The primary objectives of this study include investigating the bedrock layer and determining its depth and thickness; as well as identifying the near-surface structural features that may affect the region.

The seismic refraction technique has proven useful in defining the depth of the bedrock, since bedrock generally has a higher velocity than the overburden^15–17,19,20,75–77^. The method has also been used to detect structural features, such as faults and fractures. These features produce disturbances in the seismic velocity field so that they are detectable as discontinuities in the seismic records^8,78–88^.

## Geologic setting

**Fig. 2:**
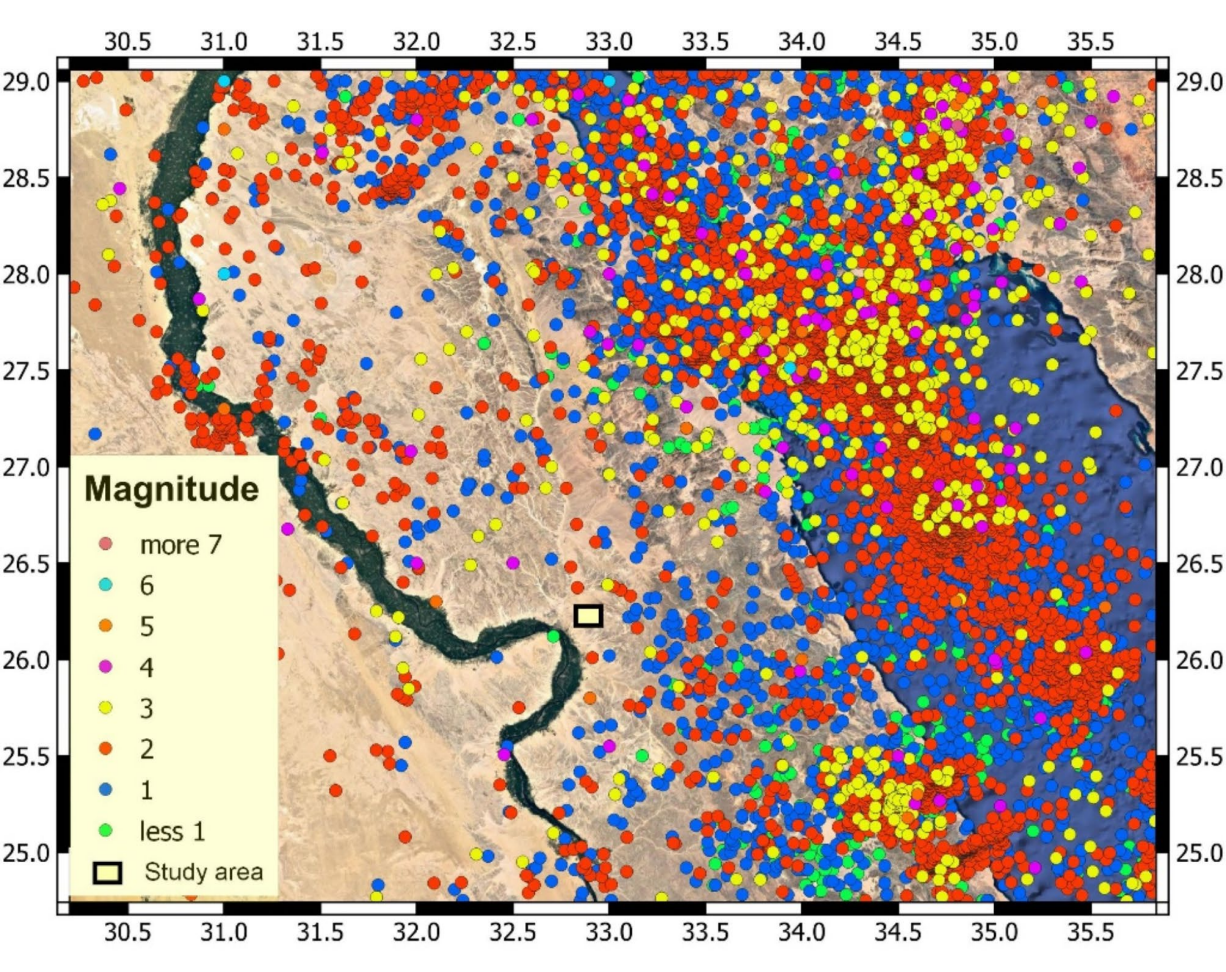
Seismicity map around Qena region based on combined catalogue from NRIAG. The yellow rectangle marks the area under investigation [Modified after^101,102^].

The New Qena City lies between two important geologic regimes in Egypt: the Qena-Safaga Shear Zone and Wadi Qena. Wadi Qena is a structural-controlled valley, in which development was undergone along faults oriented parallel to the mountain ranges of the Red Sea as well as the Gulf of Suez. These faults maintain their activity over several periods of time until recent days^48,90–92^. Figure 2 depicts a greater distribution of earthquake activity around the study area from the Gulf of Aqaba, Gulf of Suez, and Red Sea towards Nile River basin. In contrast, there are fewer and infrequent earthquakes in the west and far west^93,94^. In the same context, most of the wadis and drainage channels in the studied area are in some part, structurally controlled and developed in structural lines such as joints and faults^56,91,92^.

The Qena-Safaga Shear Zone (QSSZ) is a structural discontinuity that runs ENE–WSW and NE–SW^95^. It represents a right-lateral shear zone of Precambrian age, incorporating numerous NE-SW, N-S, E-W, and NW–SE tectonic orientations^92,96,97^.

**Fig. 3:**
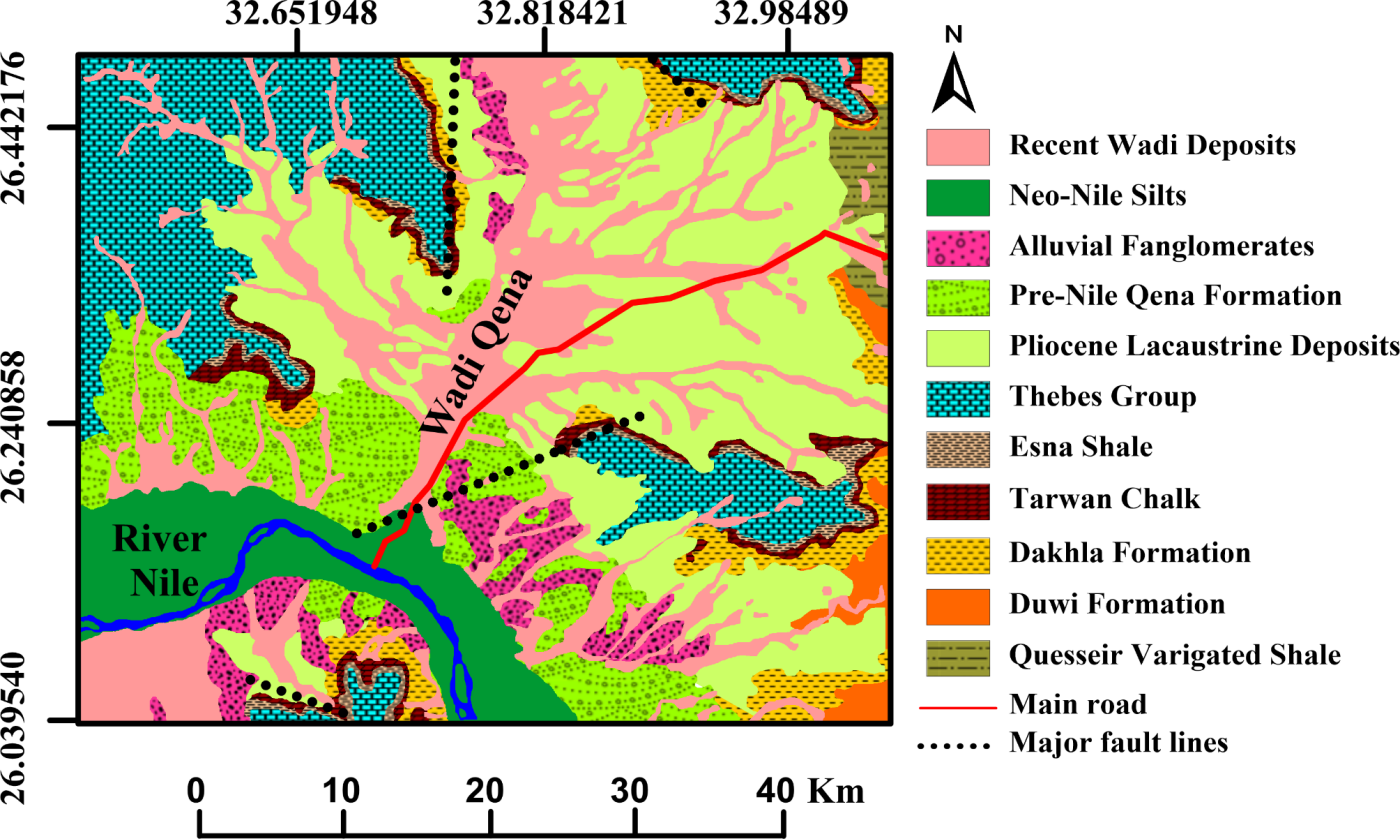
A map illustrates the spatial distribution of the geological units in Wadi Qena and surrounding areas (modified after CONOCO, 1989^62^).

The geologic units outcropping in Qena area are shown in the geologic map (Fig. 3) and include:

1. The Upper Cretaceous-Lower Eocene sequence:

From base to top, this sequence consists of: Nubia Sandstone (fine to medium sandstone intercalated by green shale, of pre-Cenomanian age), Qusseir Variegated Shale (multicolored laminated shales of Campanian age), Duwi Formation (phosphate beds interbedded with marl and oyster limestone of Maastrichtian age); Dakhla Shale (calcareous and gypsiferous shales and marls of Maastrichtian—Danian age), Tarawan Chalk (marl and marly limestone of Upper Paleocene age), Esna Shale of Upper Paleocene age, and Thebes Formation (carbonate sequence of Lower Eocene age).

2. Pliocene sediments:

The Pliocene sediments are lacustrine deposits covering the Eocene Thebes Formation and are represented by the Durri Formation^62,98^. The Durri Formation consists of rounded gravels with rolled fragments of Ostrea sp. and oolitic limestones, marls, mudstones and limestones, along with shale, sandstone and conglomerate lenses and tongues^63^. These sediments were deposited in low-energy aquatic environment suggesting substantial ancient lake systems^57^. The sediments of the Durri Formation were primarily derived from intermediate igneous rocks in southern Egypt and northern Sudan, transported by fluvial processes into the deposition sites^58^. The Durri Formation in the Qena area has a heterogeneous composition due to the lateral facies variation that characterizes the unit. The clay content of the layer contains significant amounts of illite, smectite and kaolinite. In addition, the layer is highly fissured. These features affect the geotechnical properties of the Durri Formation, which influence its behaviour in engineering and construction applications.

3. The Quaternary sediments:

The Quaternary geological units in the study area include:

A. Prenile sediments:

They are represented by Qena Formation, which consists of a thick succession of fluvial and lacustrine sediments of the Middle Pleistocene age^99^. It consists of sandstones, siltstones, and mudstones, with intercalations of conglomerate and limestone^100^. The formation’s depositional environment is indicative of a dynamic interplay between riverine and lacustrine processes, reflecting periods of fluctuating water levels and sediment supply^57^.

B. Recent alluvial sediments:

These are the unconsolidated deposits that accumulated during the Recent on the floor of the wadis and above the terraces. They include alluvial fanglomerates and wadi deposits.

**Fig. 4:**
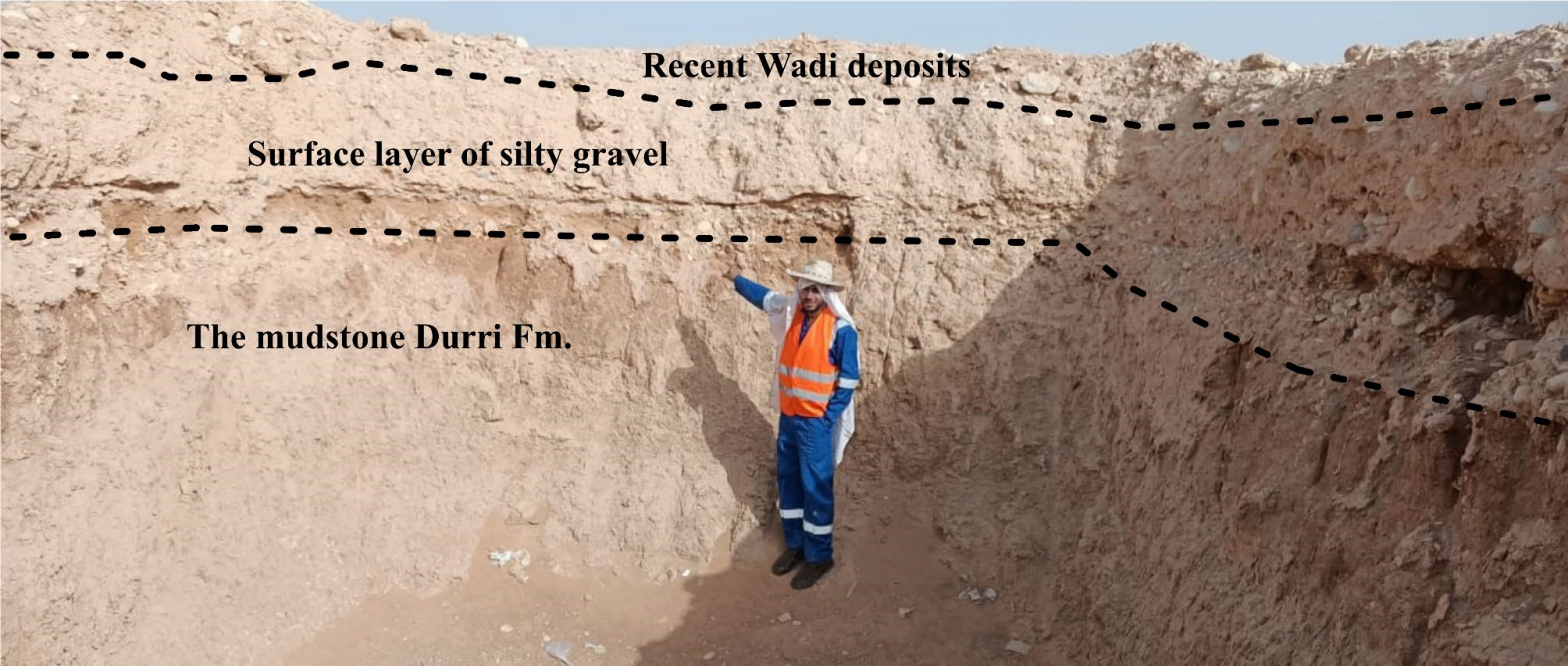
The shallow stratigraphic units revealed by a drilled borehole near line P1, highlighting the subsurface geology of the investigated area. The uppermost layer consists of a thin cover of Recent wadi deposits, primarily composed of silts, sands, and gravels. Beneath this, a silty gravel layer, ranging from 1 to 1.5 m in thickness, is present. This layer appears more silty in outcrop sections, with gravel concentrated at its base. Underlying the gravel layer is the Pliocene Durri Formation, characterized by a thick mudstone unit that extends into the subsurface.

The Pliocene and Quaternary sediments represent the shallow layers in the study area. Field observations show that the Pliocene Durri Formation is overlain by a silty gravel and less compacted weathered layer which is in turn covered by a thin bed of loose Recent wadi deposits consisting of fine sand, silt, clay, and gravel (Fig. 4). The upper part of the silty gravel is weathered and less compact. Therefore, the geologic characteristics of these layers have the most impact on the objectives of the investigation.

## Seismic refraction data acquisition

The designated region of investigation was partitioned into two sections, separated by unpaved road, that was difficult to access to acquire the seismic data. Therefore, in each section, the seismic refraction data was acquired on a group of five lines, giving a total of ten profiles for the whole area (Fig. 1). In each group, four lines were oriented NE-SW parallel to Qena-Safaga Road and perpendicular to the suspected fault locations. A fifth line, orthogonal to the other four profiles and crossing them, was acquired in the NW–SE direction.

The eight NE-SW profiles (P1 to P4 and P6 to P9) consist of three overlapping spreads. The length of each profile is 450 m, and the spread length is 180 m, with an overlap of 45 m. The two NW–SE profiles, P5 and P10, each have a length of 315 m and consist of two overlapping spreads. The length of the spreads was chosen so that a depth of investigation of around 50 m is achieved, to ensure studying the thickness of the bedrock. The average thickness of the Durri Formation as estimated from the geological studies is around 30 m^98^. The rP-waves was recorded using a seismograph with a twelve-channel system and vertical 14-Hz geophones, with a total of 36 geophones per spread. The geophone interval is 5 m which ensures desired lateral resolution. This interval is suitable for mapping subsurface geology in intermediate-depth investigations (10–100 m)^103,104^. To avoid poor coupling and false readings, the geophones are firmly planted into the ground after digging to 30–50 cm to remove loose soil. A 30 kg weight dropper was used as the energy source and shots were stacked 3 to 5 times. The source of such a weight and stacking the signals up to five times are important to increase the signal-to-noise ratio of the recorded data. The proximity to the Qena-Safaga Road introduces noise from traffic, but these methods help mitigate it. Noise levels are continuously monitored, and survey operations paused during rush hour. Real-time data review ensures signal clarity, and poor-quality readings are repeated to ensure data reliability.

For each spread, a number of seven shots were recorded which enabled adequate sampling of different layers and interfaces based on information on subsurface geology from previous investigations. The distance between shot points was 30 m which provides a good coverage of the subsurface to the desired depth of investigation, with a reasonable resolution. Acquisition parameters included a recording length of 0.5 s and a sampling interval of 0.250 ms. The short sample interval captures the highest frequency of the seismic signal, improving spatial and temporal resolution, which is crucial for accurately resolving arrival times and delineating geological boundaries. No acquisition filters were used.

## Seismic refraction data processing and inversion

**Fig. 5:**
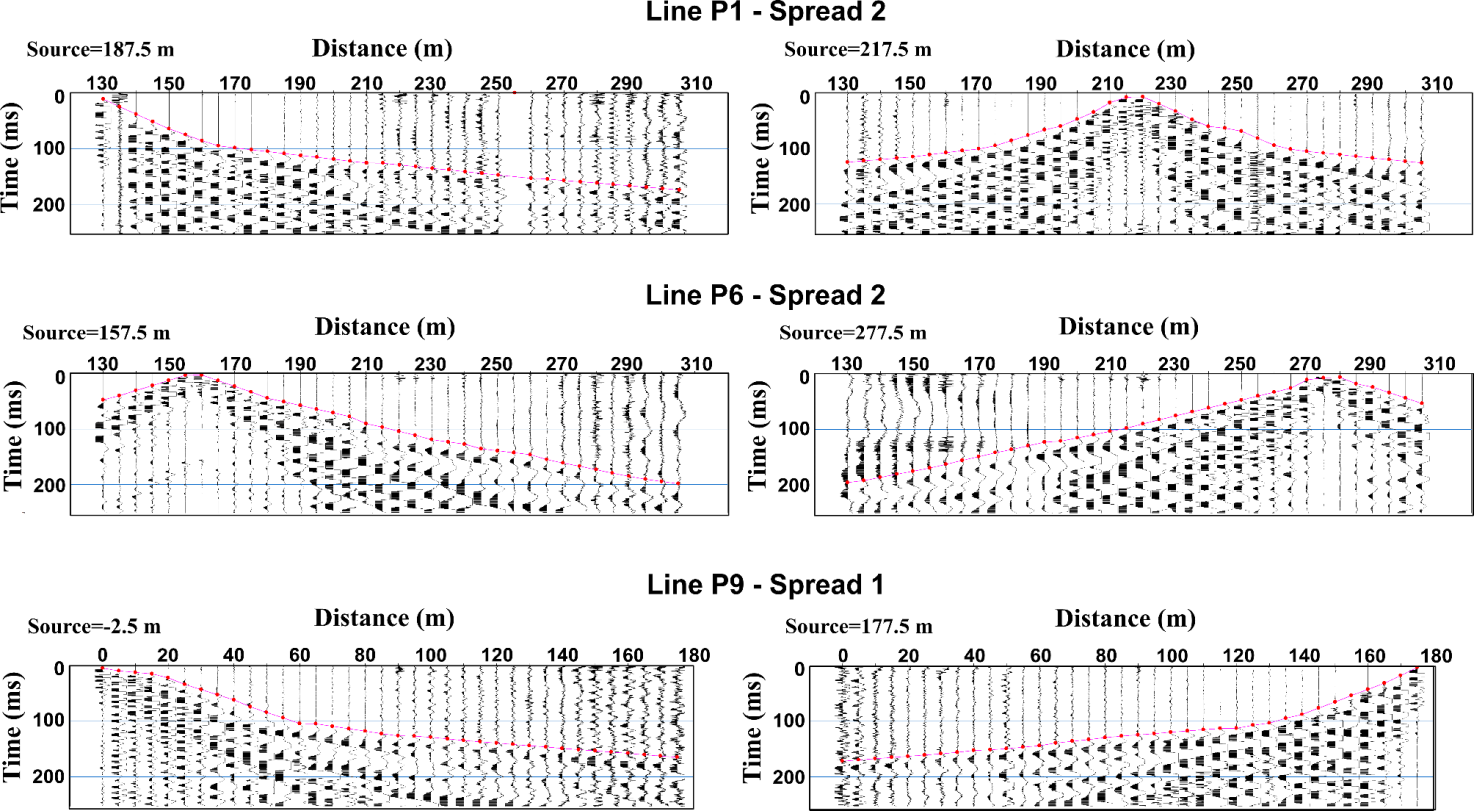
Examples of first arrival times picking of selected shots. Record length is truncated to 250 ms.

Shot and receiver distances as well as topography were assigned for all the measured shot records, based on GPS readings of all the measurement points in location. First arrival times for the recorded P-wave data were picked manually using Pickwin, which is the picking module of the SeisImager software package. The quality control procedures employed during the data acquisition, such as noise monitoring and strong geophone coupling, ensured high signal-to-noise ratio for the shot records. Therefore, a consistent picking is enabled for the acquired shots. Bad and noisy traces were killed to avoid misleading picking. The picked first arrivals were regularly reviewed and verified to maintain accuracy. Examples of picked shot records are shown in Fig. 5. A number of 1008 first breaks were picked, and then used for traveltime tomography.

The first breaks were inputted in Plotrefa, which is the inversion module of SeisImager. The first breaks were graphically represented in relation to the distances between the source and the geophones, and traveltime-distance curves were constructed and examined in order to accurately interpret the recorded data. The algorithm conducts nonlinear traveltime tomographic inversion^105,106^ to create 2D depth-velocity models. This inversion method addresses the complexities of seismic wave propagation and the inherent nonlinearity between travel times and subsurface velocities^107^. It involves forward modeling to compute theoretical travel times and inverse modeling to adjust the velocity model based on the discrepancies between observed and predicted travel times^108,109^. The initial model is constructed using another algorithm that uses the 2D homogenous function method of two coordinates to automatically invert the first arrivals to derive a 2D velocity distribution giving a local approximation of the real velocity fields^110–112^. The homogenous function method is thus very suitable as the it assumes that the approximation of real geological media corresponds to the properties of the real seismic media^113,114^. This provides enough flexibility to model the shallow complex geology.

**Fig. 6:**
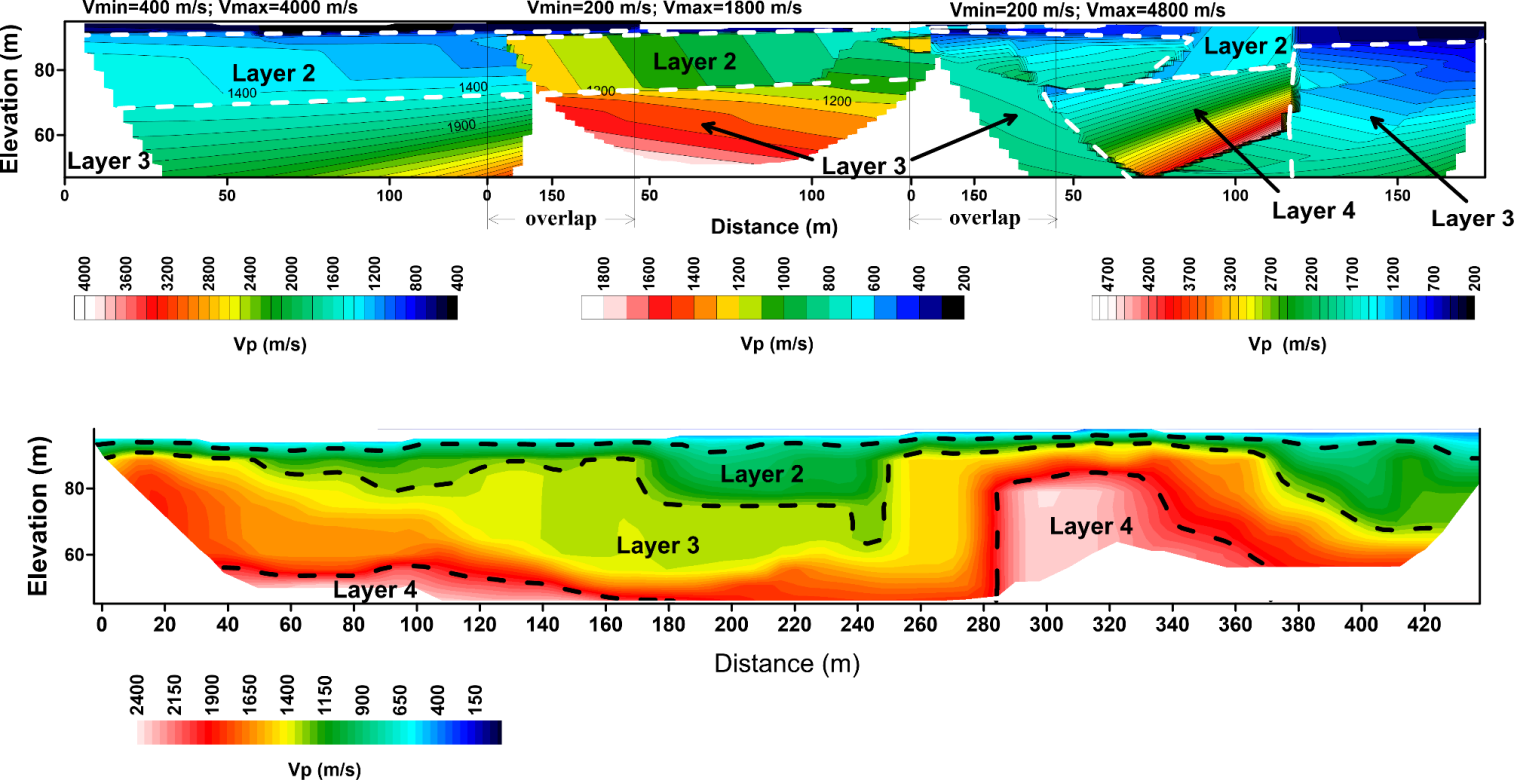
The models produced by the 2D homogenous function method (upper) that are used as an initial model to create the final tomographic model (lower) for line P1. The algorithm cannot process the overlapped spreads, so every spread is inverted independently. The generated models can be divided into layers based on the velocity gradient. The advantage of the algorithm that each layer has an inner structure, characterized by dominant values of the velocity gradient and its own contour pattern. As shown, for example, layer 2 has the lowest velocity gradient, while layer 4 has the higher gradient. The models generated by the homogenous function methods show strong correlation with the final interpreted tomographic model, and thus increases the reliability of the results.

**Fig. 7:**
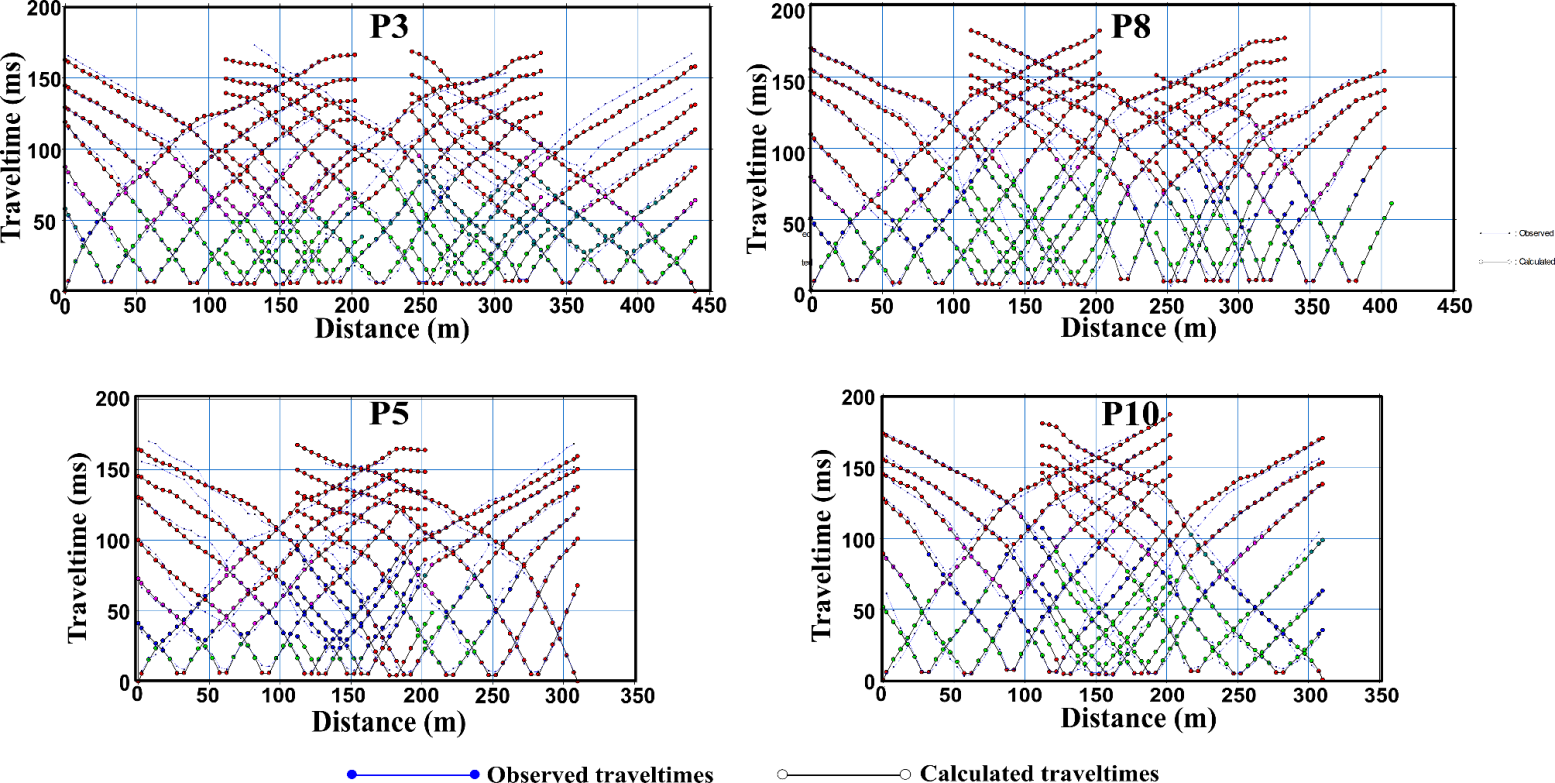
Examples of the traveltime-distance curves of selected profiles of the survey.

**Fig. 8:**
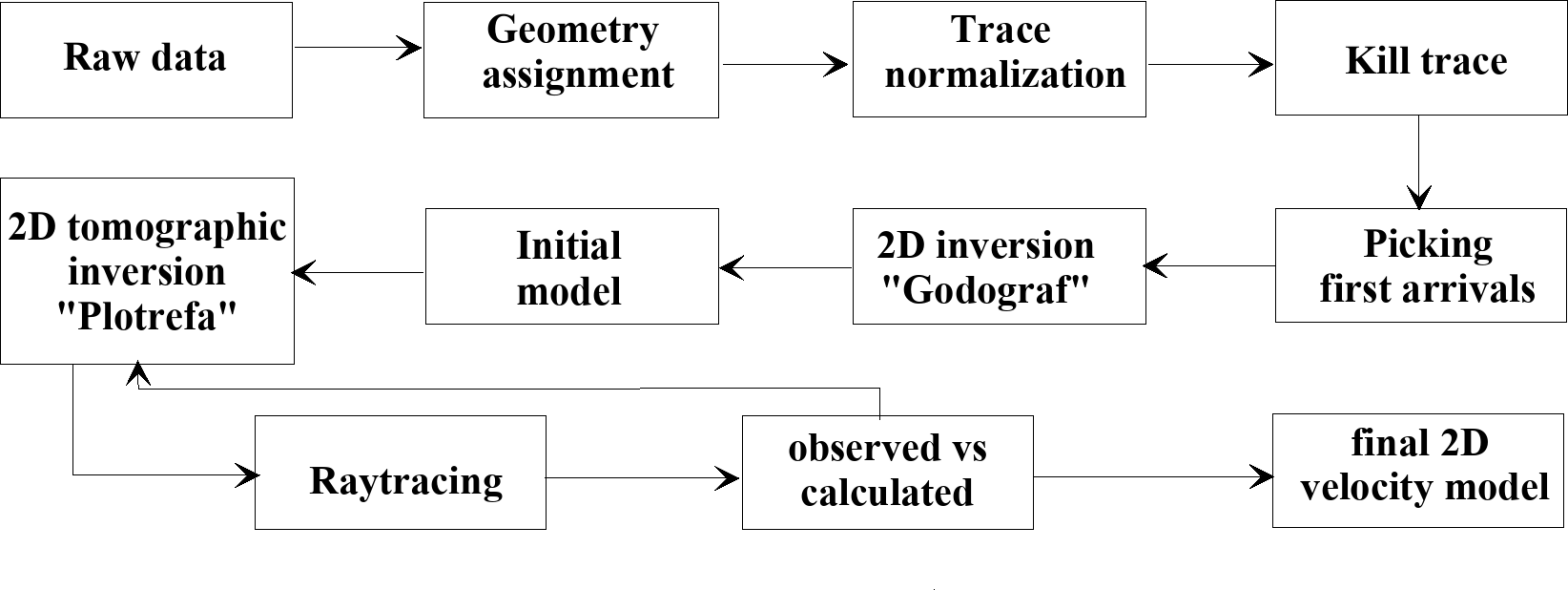
A flow diagram illustrates the steps of the seismic refraction data analysis.

**Fig. 9:**
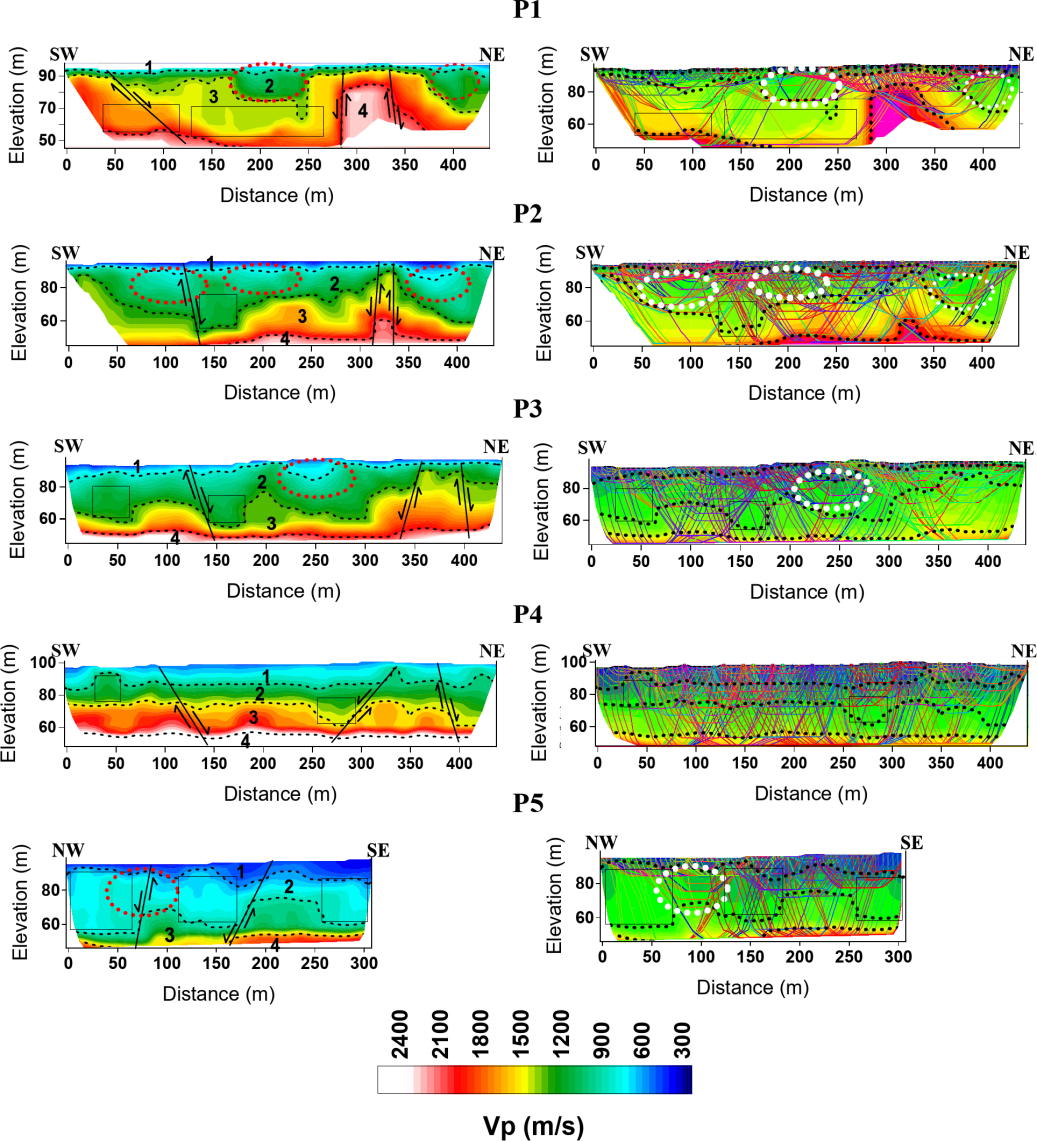

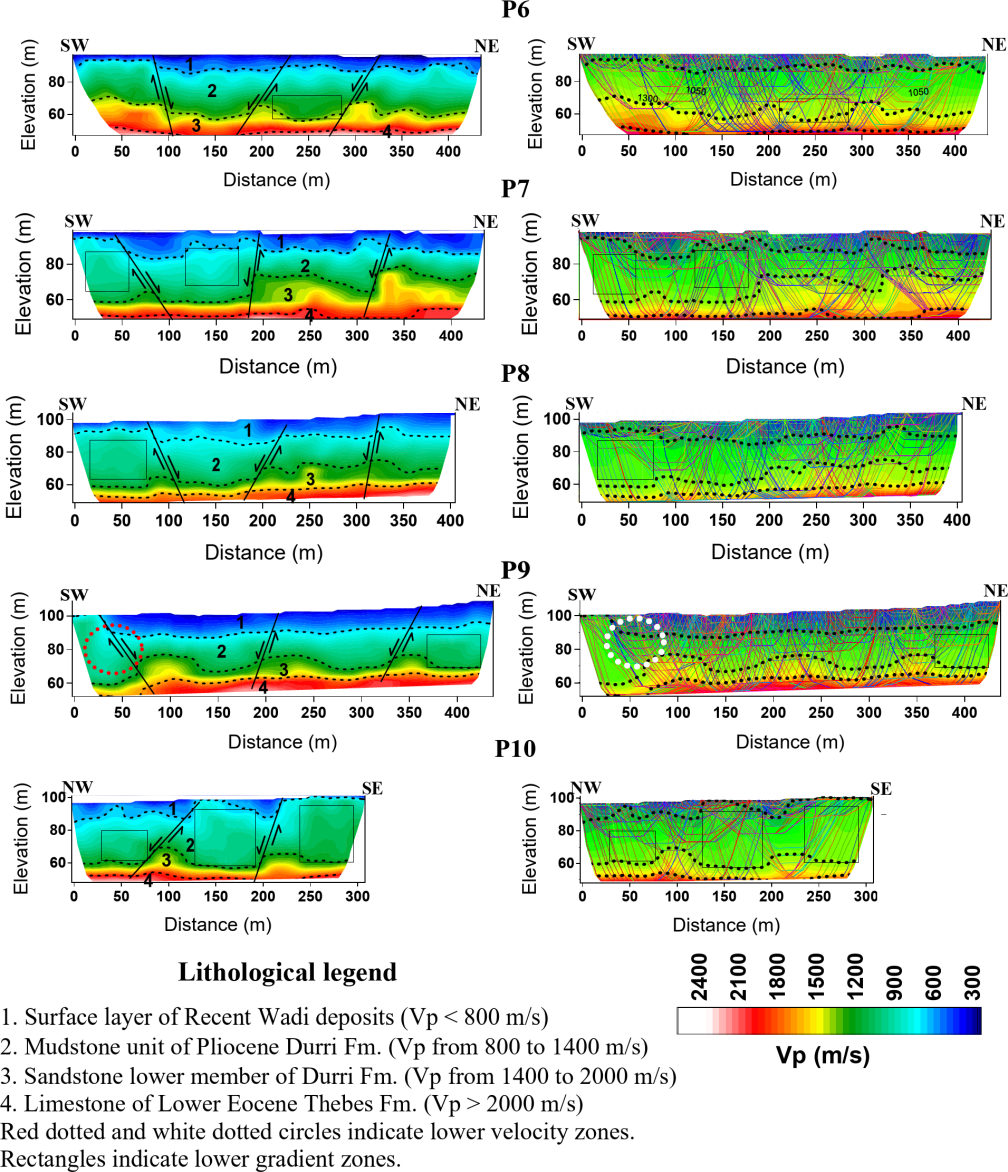
Tomographic models as well as raytracing results of the seismic refraction lines (P1 to P5) in the western part of the study area. Weakness zones could be recognized as areas with lower velocities, marked by red dotted circles on the tomographic models. These areas are also marked by white dotted circles on the ray density diagrams. Faults are recognized on the velocity sections by visible displacement of the velocity contours. Areas with low velocity gradient are marked by rectangles. Tomographic models as well as raytracing results of the seismic refraction lines (P6 to P10) in the eastern part of the study area.

Accordingly, the picked first arrivals were manually inputted into the Godograf software for traveltime inversion based on the homogenous function method of two coordinates. Determination of the combined velocity field for a multishooting geometry involves two steps: a rigorous solution of the inverse problem, which is to determine an increasing homogeneous function from two reversed traveltime curves of first arrivals, and superposition of the velocity functions calculated for different pairs of traveltime curves^81,115,116^. The final velocity section is then represented by velocity values computed at points of a rectangular grid (the grid representation). Information from the resulted models including minimum and maximum velocity, number of layers and depth to the lowest layers are used to construct the initial model for the final tomographic inversion (Fig. 6). The initial model is refined through iterations by minimizing the residuals, which are the differences between observed travel times and calculated travel times. This minimization is typically achieved through the least squares method^117^. The number of iterations is set to 10. All the recorded shots were used for tomographic modeling. An RMS error ranging from 1 to 4 ms is estimated, indicating an optimal fitting between the measured and calculated traveltimes (Fig. 7). To refine the interpretation of the models, raytracing routine in Plotrefa was performed to check the consistency between the model and the data. This can be achieved by forward modeling to estimate the penetration of the seismic rays used to calculate the synthetic traveltimes in the tomographic inversion^118,119^. This raytracing was important to verify and refine the reliability of the data at depth. Figure 8 summarizes the steps used for the inversion of the seismic refraction data. Ten two-dimensional tomographic models were produced and are shown along with raytracing results in Fig. 9.

## Results and discussion

Previous research on the Durri Formation, recognized as the bedrock from the region east of Qena City to KM 40 along the Qena-Safaga Road, has concentrated on the geotechnical issues associated with this layer, including the destruction of the asphalt road, buildings, and underground water pipelines^66,70,120,121^. Large fractures are formed in the area between KM 18 to KM 30 on Qena-Safaga Road that were attributed to the geotechnical behavior of the layer^67–69,122^. This study provides new insights into the Durri Formation, utilizing velocity measurements to enhance our understanding of its subsurface characteristics. Key findings include the previously unexamined subsurface thickness variations of the formation, the identification of fractured zones and less compacted areas within the bedrock, as well as refined lithological information. Furthermore, this research explores subsurface faulting and its implications on the area, addressing critical gaps left by earlier investigations. In addition to seismic velocity, the velocity gradient will be use in the interpretation to define the characteristics of the layer. The use of the velocity gradient proved to be effective tool in the interpretation of the shallow layers^123^.

By careful examination of the resulting depth-velocity models and comparing the results with the geology of the area from previous studies of^57,69,70,124^, four geoseismic layers were recognized. Table 1 presents the depth and thickness ranges of the geoseismic layers derived from tomographic inversion analysis. The interpretation of these geoseismic layers is explained below.

**Table 1:** The thicknesses and depths of the geoseismic layers, estimated from the tomographic models. The values of the thickness and depth represent the minimum and maximum value at each model.

Line	Layer 1 Vp > 800 m/s		Layer 2 Vp = 800—1400 m/s		Layer 3 Vp = 1400 – 2000 m/s		Layer 4 Vp > 2000 m/s
	Depth (m)	Thickness (m)	Depth (m)	Thickness (m)	Depth (m)	Thickness (m)	Depth (m)
P1	0	1–4	1–4	3–25	5–29	8- > 42	13- > 48
P2	0	2–11	2–11	4–38	6–44	10- > 30	36- > 50
P3	0	3–12	3–12	4–34	7–39	6–36	43–49
P4	0	3–14	3–14	9–26	21–38	7–22	42–47
P5	0	2–14	2–14	11–40	22–43	7- > 23	43- > 48
P6	0	3–12	3–12	19–34	26–40	7- > 18	44- > 49
P7	0	3–16	3–16	10–34	21–43	8–32	42–49
P8	0	6–16	6–16	14–35	27–40	6–16	43–47
P9	0	1–14	1–14	12–41	25–42	5- > 17	38- > 48
P10	0	1–12	1–12	19–40	28–42	6- > 16	43- > 48

The uppermost geoseismic layer is characterized by a Vp of less than 800 m/s and represents the upper surface layer, which consists of silty gravel. The layer exhibits a reduced thickness along lines P1 to P3, with an increase observed in lines P4 and P5. In the eastern portion of the study area, the layer becomes thicker, but it thins abruptly toward the southwestern side of the models.

The P-wave velocity of the underlying geoseismic layer ranges from 800 to 1400 m/s. It represents the mudstone unit of the Pliocene Durri Formation^69,125^. The thickness of this layer varies significantly across the study area. In the model of line P1, the layer exhibits its thinnest sections. Across all models from P1 to P4, a pronounced thinning of the layer is observed between the 250- and 400-m marks. In the eastern part of the study area, the layer exhibits a greater thickness in the southwestern and central portions of the models, with a decrease toward the northeast. This variability in thickness is attributed to topographical changes in the subsurface and the displacement of subsurface faults. The model of line P5 suggests that the thinning observed in line P4 is likely due to fault displacement in the region between lines P3 and P4.

**Fig. 10:**
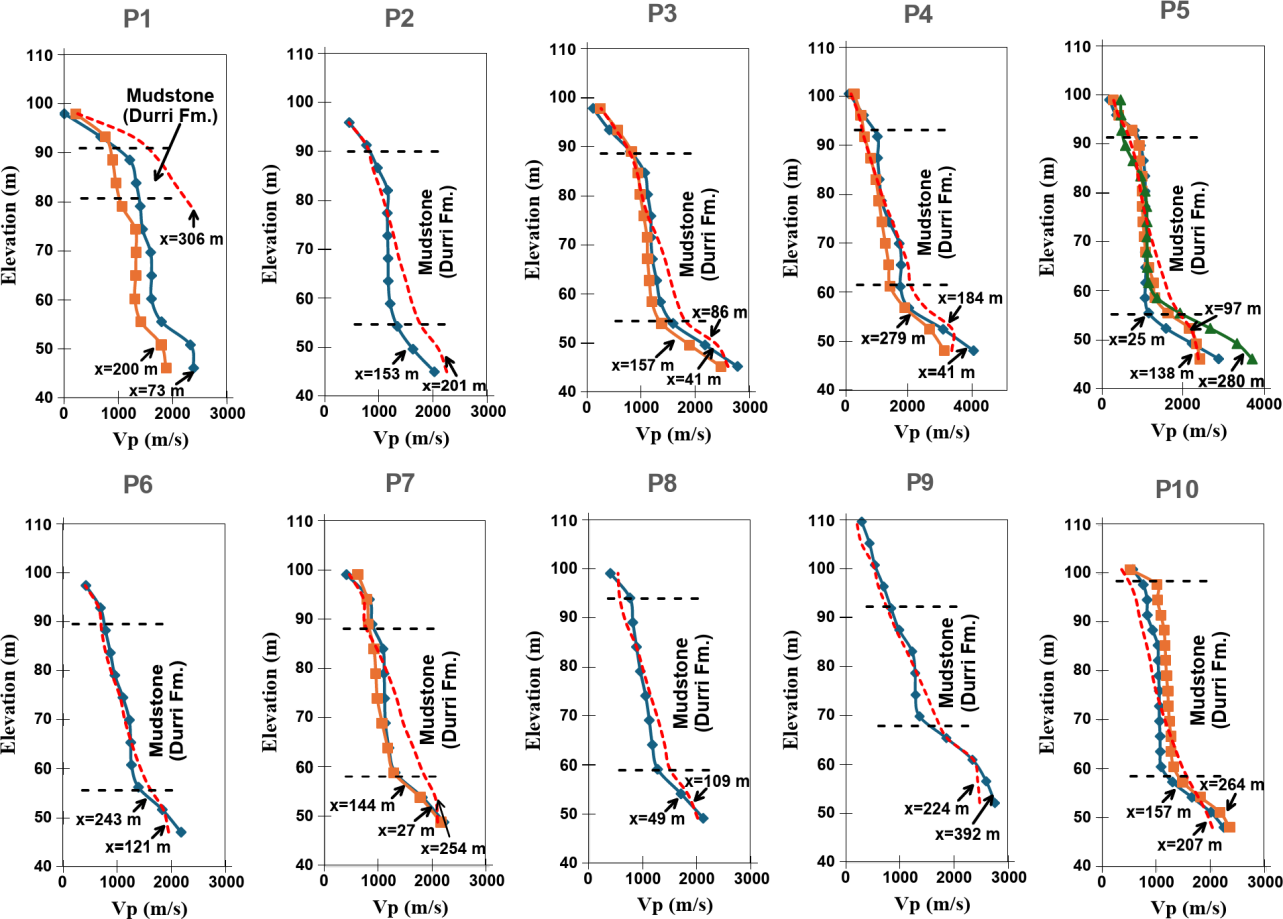
Vertical velocity profiles at midpoints of the low velocity gradient zones (rectangles in Fig. 9) along each line. Note that at these locations the curves have the steepest gradient at the mudstone layer compared with the overlain and underlain layers. The red dashed curves are arbitrary vertical velocity profiles at location where velocity increased smoothly with depth, i.e., areas with high velocity gradient. Also, note in line P2 that the layer starts with high velocity gradient then at the elevation mark 80 m, the gradient begins to decrease. The same is noted for line P9.

The velocity gradient within this layer, as reflected in the models, is notably variable, where it exhibits low, medium and high velocity gradients in the vertical and horizontal directions. This variability suggests significant heterogeneity in the material properties of the layer, which may result from differences in lithology and compaction^126–130^. Geological and petrographic studies of the Durri Formation the Qena-Safaga district^69,131^ indicate a lateral facies change in the layer. The formation consists of various sedimentary facies, each exhibiting distinct lithological characteristics. The presence of sand and conglomerate intercalations within the layer, observed in the outcrops further supports this heterogeneity. Areas with low velocity gradient are marked by rectangles in the models. In raytracing models, these areas are characterized by medium to low ray coverage. This is because when seismic waves encounter these zones, the waves slow down, which causes their paths to bend. This bending occurs due to the contrast between the slower and faster zones, leading to changes in the angle of refraction according to Snell’s Law. Ray paths will bend towards the normal as they enter a slower medium and away from the normal as they exit into a faster medium^132–134^. In the models of lines P1, P5 and P10, these areas extend in most of the section while occupying less zones in the other lines. According to^43,123,135^, low velocity gradient reflects zones of low compaction. A study of the vertical velocity gradient in these areas is presented in Fig. 10. It is noted that at these locations, the mudstone layer exhibits an increase of the velocity with depth but at slower rates giving rise to steep gradient, while the upper layer and the underlying layer shows a gradual high rate of increasing of the velocity. In areas with high velocity gradient, the velocity increases gradually with depth in approximately constant rates giving smooth curves (red dashed curves in Fig. 10). According to^123,136^ this trend of the low gradient profiles refers to cohesionless rock.

The low velocity range observed in this layer can be attributed to specific geologic characteristics of the study site. In the models of lines P1, P2, P3, P5, and P9, low-velocity zones penetrating approximately 15 m into the layer are identified, marked by red dotted circles in Fig. 9. These zones are characterized by low velocity gradients. In the corresponding raytracing, these areas are marked by white dotted circles, indicating medium ray coverage. These low-velocity zones are interpreted as fracture zones, which are a known characteristic of the Durri Formation^69^. The presence of fractures and high porosity are significant contributors to the reduction in P-wave velocity^103,137–142^. Seismic rays in low-velocity fractured areas demonstrate slower travel times, increased ray density, ray bending, amplitude attenuation, and delayed arrival times^143,144^. These features were helpful in identifying and mapping the extent of the low-velocity zones within the layer. Moreover, geological studies^57,68,69^ indicate that the layer contains a significant amount of clay, which also contributes to the reduction in P-wave velocity^129,142,145–148^. The combination of these factors likely explains the pronounced lateral velocity variations observed within the layer and its overall low velocity range.

The third geoseismic layer has a Vp of 1400 to 2000 m/s, and corresponds to a marl layer that is also belonging to the Durri Formation^69,124^. This section of the Durri Formation exhibits a significantly higher velocity gradient across all models, except for line P1. This distinct behavior contrasts with the overlying mudstone layer, despite both units being part of the same formation. The increased velocity and the high velocity gradient suggest that the marl layer is more compacted compared to the mudstone. Additionally, ray coverage analysis reveals refractions at the interfaces, which have been identified as the upper and lower boundaries of this unit. Due to these factors, the marl layer is classified as a separate and distinct unit within the formation.

This layer exhibits significant depth variation across the study area, where it appears shallower along line P1 compared to other sections. A notable reduction of the depth is observed between the 300 to 350-m mark in lines P1 to P4. In the eastern part of the study area, the layer is relatively deeper, with a decrease observed in the central sections. The thickness is also variable through the sections. In the eastern portion of the study area, the layer is thinner than the western portion, with greatest thickness recorded in line P1. These depth and thickness variations are likely the result of localized uplift caused by subsurface faulting detected within the region. In line P1, the layer exhibits notable low velocity gradient that also may refer to that the layer also suffering less compaction in this part of the study area.

The fourth geoseismic layer occurred deeper in the eastern section of the study area than the western section. The layer occurred at shallow depths between 300 to 350-m mark in lines P1 to P4. This abrupt decrease in depth is attributed to the local uplift caused by two faults. The P-wave velocity within this layer exceeds 2000 m/s and is characterized by high velocity gradient. It corresponds to the limestone belonging to the Lower Eocene Thebes Group^69,70,122,124,149^.

Ten subsurface faults were detected in the velocity sections, which could be recognized by the abrupt displacements in the velocity contours. Due to the displacements of these faults, the models show apparent discontinuity in the depth of the layers, as well as variations in their thicknesses. In the ray coverage sections, the faults created limited-scale shadow zones where direct seismic waves are not detected because they are refracted at the edges of the displaced layers away from that area, leading to gaps in seismic data^150,151^. The effect of these detected faults is summarized as follows.

In lines P1 to P4, the fault in the west coincides with the one detected in the geomagnetic studies of^49,52,56,73,74^. The two faults located between 300 and 350 m along the lines, caused a sudden uplift, leading to a decrease in the thickness and depth of the second and third layers. The two faults detected in line P5 changed the thickness of the Durri Formation and the depth of the underlying limestone between lines P1 and P2, and lines P3 and P4. In lines P6 to P9, the southwestern fault reduced the thickness of the surface layer to the southwest. In line P10, two faults were observed, causing notable variations in the layer’s depths and thicknesses in the perpendicular direction. The change in dip direction of the faults along these lines indicates that the faults probably exhibit more than one movement, which is a result of the ongoing tectonic activity in Qena-Safaga district^124,152^.

## Conclusions

To estimate the depth and thickness of the bedrock and to detect the structural features, shallow seismic refraction data were measured along 10 profiles in the New Qena City extension south of Qena-Safaga Road. Four geoseismic layers were recognized from the tomographic sections. The surface layer has Vp < 800 m/s and composed of silty gravels. The second geoseismic layer is characterized by Vp ranging from 800 to 1400 m/s, representing the mudstone unit of the Pliocene Durri Formation. It has a variable thickness of about 3 to 40 m and occurred at a depth from 1 to about 16 m. The third geoseismic layer is characterized by Vp of 1400 to 2000 m/s and represents marls of the Durri Formation. It has varying depths of about 5 to 42 m and a thickness of 8 to more than 40 m. The fourth geoseismic layer has a P-wave of more than 2000 m/s and corresponds to the limestone of the Thebes Group. It occurs at depth from 13 to more than 50 m. Ten subsurface faults were also detected in the western section of the investigated region, which could be the reason for the variable thickness and depths of the subsurface layers.

Engineering considerations for urban projects focus mainly on the shallow depth for foundations. This includes the surface weathered layer and the bedrock. The surface layer in the area, as mentioned earlier is silty gravel with variable thickness throughout the area. The depositional characteristics of this layer indicate a fluvial origin, which further influences its geotechnical properties^153^. The layer’s low velocity suggests a tendency to amplify seismic waves, increasing the risk of structural damage during earthquakes, while also indicating a potential for liquefaction under saturated conditions^154^. Additionally, the fine-grained silt within the gravel may impede drainage, leading to surface water accumulation, particularly in low-lying areas^155^.

The mudstone Durri Formation layer is considered the bedrock in east Qena region. Based on the results of this research, this layer exhibits a low range of P-wave velocities due to high degree of fracturing and higher clay content. Low velocity zones are also noted in some parts of the layer. They are interpreted as fracture zones representing weakness zones in the layer. In addition, some parts of the layer exhibits low velocity gradient. These parts are interpreted as areas of low compaction compared with other parts of the rock. The layer is also characterized by lateral facies variations that cause strong lateral velocity variations in addition to fracturing and clay content. These results give rise to critical geological and geotechnical implications.

The study confirms the problematic nature of the layer^57,66–70,124,131^ based on the above mentioned characteristics. Previous geological studies indicated that this layer could undergo swelling as a result of its significant clay-rich composition. They also confirmed the presence of extensive fracturing within the layer where large-scale cracks have been formed. These fractures caused damage to the Qena-Safaga asphalt road, buried water lines and man-made structures in the sector from KM 18 to KM 32 along the road, which is in close proximity to the study area. The research area may soon be affected by these cracks, which are still forming. The significant fracturing increases the risk of landslides and slope failure, particularly during periods of heavy rainfall^156^, especially that the area is suffered from flash flood originating in the Red Sea highlands^55,60,157^. The clay content and degree of fracturing also indicate that the layer may have weak mechanical properties, leading to potential problems for infrastructure development. Additionally, the recorded lower velocity makes the layer potentially more susceptible to deformation under load. Hydrogeologically, the fractured nature of the Durri Formation enhances its porosity and potential for groundwater flow, although the clay-rich sections may limit permeability. This creates a complex and heterogeneous aquifer system in the area^60,158^, where water movement is primarily controlled by fracture networks, despite the low permeability of the mudstone matrix^155^.

The measured P-wave velocity and fracture density of this mudstone layer suggest that it could amplify seismic waves, increasing the risk of damage during earthquakes^159^. Fractured mudstone is particularly vulnerable to deformation during seismic events, which could lead to surface ruptures^160^.

Since the layer is relatively thick (in some places it reaches a thickness of about 40 m), the authors recommend avoiding construction in this part of the area or to use modern engineering techniques for safe construction. The presence of probable faults with considerable displacements detected in the velocity sections of the measured lines also supports this point of view. The detected faults indicate significant tectonic activity suggesting that the region has undergone significant deformation driven by regional tectonic forces. The study area is situated within the Qena-Safaga Shear Zone, an active, ENE-oriented, high-strain shear zone that has experienced multiple phases of fault reactivation. This ongoing tectonic activity highlights the potential for continued deformation processes in the present day^91,92,95,161,162^. These faults also raise concerns about potential seismic hazards, as their displacements can be associated with significant seismicity, which already recorded in the area^93,163–167^.

Furthermore, these faults influenced surface geomorphology in the area, contributing to the formation of fault scarps and ridges, which in turn could affect drainage patterns and erosion processes^168^. In addition to fractures, these faults act as conduits for groundwater. Fracture and fault systems in the study area influence groundwater flow by enhancing secondary porosity and hydraulic conductivity, which boost well yields^169^. These features also aid in groundwater recharge and discharge, especially at intersections of faults with active channels and paleodrainage^170^.

The research also proved the efficiency of the shallow seismic refraction method for bedrock investigations. Information based on the P-wave velocity could give valuable insights on the characteristics of the bedrock. The use of the raytracing also proved effective in the identification of upper and lower boundaries of the detected layers as well as the presence of the fractured zones and areas of low compactions, based on ray coverage characteristics. Additionally, the velocity gradient could be used as effective interpretation criteria.

## Data Availability

The data generated and analyzed during this study are available from the corresponding author upon reasonable request.
